# Including the public in pandemic planning: a deliberative approach

**DOI:** 10.1186/1471-2458-10-501

**Published:** 2010-08-19

**Authors:** Annette J Braunack-Mayer, Jackie M Street, Wendy A Rogers, Rodney Givney, John R Moss, Janet E Hiller

**Affiliations:** 1 Discipline of Public Health, School of Population Health and Clinical Practice, University of Adelaide, Level 9, 10 Pulteney Street, Adelaide, SA, 5000, Australia; 2 Department of Philosophy & Australian School of Advanced Medicine, Macquarie University, Sydney, NSW, 2109, Australia; 3 Microbiology and Infectious Diseases, Hunter Area Pathology Service, John Hunter Hospital, Lookout Road, New Lampton, NSW, 2305, Australia; 4 Adelaide Health Technology Assessment, University of Adelaide, Level 3, 122 Frome Street, Adelaide, SA, 5000, Australia

## Abstract

**Background:**

Against a background of pandemic threat posed by SARS and avian H5N1 influenza, this study used deliberative forums to elucidate informed community perspectives on aspects of pandemic planning.

**Methods:**

Two deliberative forums were carried out with members of the South Australian community. The forums were supported by a qualitative study with adults and youths, systematic reviews of the literature and the involvement of an extended group of academic experts and policy makers. The forum discussions were recorded with simultaneous transcription and analysed thematically.

**Results:**

Participants allocated scarce resources of antiviral drugs and pandemic vaccine based on a desire to preserve society function in a time of crisis. Participants were divided on the acceptability of social distancing and quarantine measures. However, should such measures be adopted, they thought that reasonable financial, household and psychological support was essential. In addition, provided such support was present, the participants, in general, were willing to impose strict sanctions on those who violated quarantine and social distancing measures.

**Conclusions:**

The recommendations from the forums suggest that the implementation of pandemic plans in a severe pandemic will be challenging, but not impossible. Implementation may be more successful if the public is engaged in pandemic planning before a pandemic, effective communication of key points is practiced before and during a pandemic and if judicious use is made of supportive measures to assist those in quarantine or affected by social isolation measures.

## Background

The emergence of a novel human influenza A (H1N1), early in 2009, saw the implementation of national pandemic influenza (PI) plans around the world. These plans had been developed, at the urging of the World Health Organisation (WHO), partly in response to the emergence of a virulent Avian Influenza A (H5N1) and partly in response to the 2003 outbreak of Severe Acute Respiratory Syndrome (SARS), both of which demonstrated gaps in global and local responses to infectious, clinically severe respiratory diseases.

National plans [1] place considerable emphasis on stockpiles of antiviral drugs and prototype vaccines for H5N1 strains. A second significant aspect of national pandemic planning is the use of isolation, quarantine and social distancing measures [2]. These measures, along with heightened hygiene compliance, appear to be amongst the very limited tools available to buy the time needed to produce and distribute an effective vaccine [3-5]; and as such have been employed in many countries, albeit in a limited way, in response to the influenza A (H1N1) 2009 pandemic.

The 2009 WHO Guidance document on Pandemic Preparedness [1] emphasises the need for a whole of society approach to pandemic planning. In particular, it emphasises the important role of civil society organisations, families and individuals for an effective response to a pandemic. A number of strategies to include citizens in planning and to test the public acceptability of policy measures have been used. These include community surveys [6-9], broad community engagement processes [10], and deliberative forums [11].

Each strategy has advantages and disadvantages. Surveys can inform policy decision-making through providing information about likely or actual public responses to policy initiatives. For example, surveys can tell us something about whether a community is likely to respond cooperatively to a school closure. However, a legitimate concern in gauging community views in this way is that understanding in the community about complex areas of healthcare planning and policy may be weak. This may be particularly the case for new health threats (such as a pandemic) and complex health policy areas (such as decisions about who should have priority access to scarce pharmaceuticals).

This methodological weakness may also apply to qualitative methods such as focus groups or interviews. These approaches can tell us more about the *reasons *that underpin a particular community response. However, qualitative methods will still suffer from the criticism that community perspectives in this area are of greatest value if accessed at the time of a health emergency when there is likely to be more interest in and access to information. Regardless of whether surveys or qualitative methods are used, the general lack of knowledge in the community about the emergence, transmission and management of respiratory epidemics may be a drawback in collecting community views about pandemic planning. In addition, policy makers may question the validity of citizen-driven decisions when the citizens have limited access to information to adequately inform those decisions.

For this reason, deliberative processes have been proposed as an anticipatory tool which can gauge the views of the community in policy areas that may generate future issues but where there is little current public debate [12]. Deliberative methods involve prolonged engagement with community members and provision of detailed information which the participants may draw upon in their decision making. The theory underpinning deliberative inclusive processes is that, given enough information about a topic, a small representative sample of the population can deliberate with conscience and arrive at a decision which is informed, but also reflective of community values. Deliberative inclusive processes such as citizens' juries and deliberative forums have been used for looking at issues about health service provision and priority setting in health [13-15] and less commonly for more complex policy issues such as: biobank management [16,17], mammography screening criteria [18], organ donation shortfalls [19], and genetic testing debates [20,21]. The community perspectives provided can support the development of policy that is both responsive to community concerns and which recognises the importance of community values and beliefs in policy formation but they also fulfil a loftier ideal of devolved democratic decision making [22].

In this paper, we describe the findings from FluViews - a community consultation project held in 2008 that elucidated community perspectives on some of the strategies proposed for pandemic planning in Australia. The paper describes the findings from two deliberative forums: the first explored the allocation of scarce pharmaceuticals in a pandemic; and the second examined more complex questions about the use of social distancing measures and quarantine restrictions.

## Methods

Two deliberative forums were conducted in Adelaide in 2008 to consider the following questions:

Forum 1: Who should be given the scarce antiviral drugs and vaccine in an influenza pandemic?

Forum 2: Under what circumstances would quarantine and social distancing measures be acceptable in an influenza pandemic?

Forum 1 met for one-day whereas forum 2 was over 2 days. Only forum 1 and the second day of forum 2 are reported here. Other findings from forum 2, relating to effective communication in a pandemic, and details of the methods used, have been published previously [23].

Both forums used aspects of the citizens' jury model including: a steering group of academic experts and policy makers; random selection of participants to reflect the population; delivery of information in an interactive, accessible and non-threatening way; facilitation by an independent facilitator; participant deliberation in small groups and as a whole; and the formulation of a 'verdict'. The process differed from some models of citizens' juries in that the forums were smaller and shorter. In addition, we analysed the transcripts of the deliberations using thematic analysis (methods described previously in [23]) to elucidate further the underlying reasons for the choices made and ensure that minority opinion was included. We have used pseudonyms to identify participant quotes.

### Preparation for the Forums

Four focus groups - two with adults and two with schoolchildren aged 16-17 - were used to determine the main issues of concern and help frame suitable questions for the forums. School children were included in this process because many social distancing measures will adversely impact on young people and because this group can be important vectors in the spread of disease [24] as demonstrated in the current swine flu outbreak. Supportive documents for the specialists and the forum participants were based on systematic reviews summarising available evidence about pandemic influenza containment and management strategies. Policy makers working in pandemic influenza planning engaged with project development throughout.

### Recruitment

Difficulties were encountered in recruiting enough participants for forum 1: withdrawal for a variety of reasons led to a forum with 9 participants, which was older and with more females (n = 6) than males (n = 3). Forum 2 had a full quota of 12 participants. Other than age and gender the distribution of demographic identifiers for forum 1 was similar to that for forum 2 (described in [23]). Honorariums of Aus$100 and Aus$300 were paid to each participant in forums 1 and 2 respectively.

### Assumptions

In both forums, the participants were asked to assume that the influenza virus would cause moderate case morbidity across age groups and moderate case mortality although the jury was also told that in pandemics, the usual epidemiology of flu may be reversed, so that young adults get more disease than the elderly. The forum was provided with case figures based on mathematical modelling of a projected pandemic in South Australia (G. Tucker, Department of Health, Government of South Australia, Personal Communication) using the FluAID software package [25] which remain consistent with WHO planning recommendations [1]. In forum 1 it was assumed that influenza infection was geographically widespread and that initially there would be sufficient antiviral drugs from the national and state stockpiles to treat approximately 10% of the population or provide prophylaxis to 5%. In forum 2, the participants were asked to deliberate using a series of six scenarios which sketched a range of situations in which quarantine and social distancing measures might be used (Table 1). There were opportunities for the participants to engage with the experts at all stages of the deliberation and discussion.

**Table 1:** Scenarios used with forum 2

Order	Scenario	Should quarantine/social distancing be required?
1	Miriam has recently returned from a ten week work placement in an infected country. One person on her flight developed a fever on the flight, but it is not yet known if the person is infected with the pandemic strain of influenza. Miriam has been asked by the health official at the airport to remain at home in quarantine. Miriam's husband is still travelling and Miriam knows there is no food in the house.	Miriam should comply, provided she has enough support at home.

2	South Australia is in week two of the pandemic. Miriam died yesterday. Her husband David has rushed home from aboard and was at her bedside when she died. He has been issued with a formal order to remain in quarantine for the next seven days. Miriam and David's religion requires that the body be buried within 24 hours. Miriam had a large extended family, was very active in the community and had many friends but David has been told that only the immediate family can attend the funeral.	Forum divided on whether attendance at funeral should be restricted (including whether David should be prohibited from going).

3	Kimberley gets a call to tell her that Rob has been diagnosed as ill with the pandemic strain of influenza. Kimberley and Rob had lunch together at a hotel yesterday. At the time he complained of not feeling well and said he would be visiting the doctor that afternoon. Kimberley is very worried that she has been exposed. She knows that if she goes into quarantine voluntarily she will be unable to work for up to seven days and it may be longer if she becomes ill. She works for a small bakery franchise and is aware the manager is already having staff problems. If she does not turn up for work today and tomorrow, the store may not open. Kimberley is a single parent has no savings and is totally reliant on her income from the bakery for daily living expenses. She is anxious about infecting her children if she continues to live with them, but she has no alternative sources of child care.	Forum divided: a. Kimberley should seek more information about obligations (with a view to going into quarantine) b. Kimberley would/should just go home (and pretend nothing has happened)

4	There have been only a small number of influenza cases in South Australia but several hundred in Sydney. The Premier is considering closing schools. Year 12 exams are in five weeks.	Forum divided: a. Schools should be closed b. Not enough evidence yet to close schools

5	Margaret, Wilma's daughter is the chairperson of a rural school council. The Premier has closed all schools and childcare services in the State just when farmers in the area are sowing their annual crops. Margaret realises that many farming families will not be able to care for their children and sow the crops. She decides to organise a home based community childcare service. Families are placed on a roster to provide care for up to 15 children each day. The arrangement continues for about four weeks until the busiest part of the sowing season is over.	Forum divided: a. Make an exception to social isolation measures in certain situations such as these b. No exceptions; childcare arrangements should be closed down.

6	Wilma has gone to church nearly every Sunday for most of her life and she has begun attending on weekdays. She has heard that the Premier has closed schools and banned all public gatherings, including club meetings and church services. Wilma is very anxious about missing church. On the other hand, she is also worried about catching flu.	Forum divided: a. Ban all social gatherings, including religious services b. Religious gatherings should be allowed to continue

### Post-forum evaluation

Participants were contacted by telephone within four weeks of the forum and asked to comment on the process, the interaction with the specialists and whether they agreed with the consensus findings of the forum in which they had participated.

This study was approved by the Human Research Ethics Committee of the University of Adelaide (H-176-2006) and for the student focus groups by Community and Tertiary Liaison, Department of Education and Children's services.

## Results

### Forum 1: Prioritisation of allocation of scarce resources

The initial list of potential recipients identified was broad and eclectic and included:

health care workers; researchers and laboratory staff dealing with pandemic influenza; military; essential services (water, power, waste etc); people aged 2-30; police and prison staff; paramedics and emergency response personnel; primary producers and food transport workers; communication workers; clergy; parents and care workers, funeral organizations; decision-makers; asylum seekers; prisoners; and tourists.

Reasons for including these groups varied (Table 2) but generally fell under three broad themes: groups that would be in high demand for their services in a pandemic (health care workers, funeral organisations, emergency response); groups that were essential to the continued maintenance of societal function (essential services, primary providers, food transport workers) and vulnerable populations to which society owed a duty of care (young people aged 2-30, asylum seekers, prisoners, tourists). Several participants included people aged 2-30 years because this group was regarded as highly socially interactive and seen as both at greater risk of infection and an important conduit for the rapid spread of influenza throughout the population. In addition, some groups were selected for more than one reason. For example, young people aged 2-30 years were also included because, as one participant said, "*They are the future*."

**Table 2:** Ranking and rationale for groups to receive antiviral drugs and vaccine in a severe pandemic

Rank-Sector	Rationale	Typical Supporting Quotation
1^st ^- Health care workers	Essential to provision of medical services for PI and non-PI patients	"[They] need to administer the medicine to people, and also, obviously, see to any other illnesses and sicknesses that are going on together at the same time."

2nd - Vaccine and antiviral drug production workers	Ensuring the timely supply of developed vaccines and drugs	"If we don't have any of those guys around and they get sick, no vaccine gets developed; we are in big trouble."

Equal 3^rd ^-Essential services	Maintenance of fabric of society	"The essential services have to keep the society running. When there is no electricity, water etc the whole system will collapse"

Equal 3^rd ^- Military	Multi-skilled and experienced in disaster management	". . . transportation of fuel, *et cetera*, and any other things that might come along like that mobile hospitals and that sort of stuff."

PI = pandemic influenza

The forum quickly realised that the numbers in these groups were more than could possibly be covered by the limited stockpiles then available. In refining the lists, the forum discarded vulnerable groups such as asylum seekers and those groups considered peripheral to the preservation of society. They also removed groups such as the clergy and funeral organisations whose roles might potentially be covered by others. Some groups, such as prisoners, were considered to be at lower risk since their incarceration might be considered a form of social distancing. Most of the participants did not distinguish between antiviral drugs and vaccine, and felt distribution patterns should be similar for both.

In constructing the lists, the forum participants acknowledged that they would not benefit themselves from the choices they made. They explicitly excluded the elderly and the chronically ill from the list because they felt that the elderly were "*not the future*", would be "*more drain than help*" for an already "*depleted society"*. Some participants expressed the opinion that elderly people would prefer that a younger person have priority access to the vaccine or antiviral drug.

ROSEMARY: If you asked every elderly person whether they thought they should have it or their family, they would say their family.

In rank ordering the list, deliberations focussed on preservation of society in a time of crisis. The final choices, in order, and quotes from participants summarising the forum's reasoning are shown in Table 2.

Protecting the health of health care workers was seen as a way of protecting the well-being of all, since health care workers would be needed not only by those requiring health care services for treatment for the effects of influenza infection, but also for more routine medical needs. In addition, health care workers were seen as essential in the roll-out of the vaccine once developed:

STEPHANIE: I think the main reason I put it [health care workers] was so they would be there. People could come to the hospitals to be vaccinated.

Vaccine and antiviral drug production workers were considered essential and irreplaceable and to be so small in number as to be a relatively small drain on limited resources. The other choices were considered indispensable to preserve social structure and order by maintaining essential services. If forced to choose between preserving society in the long run and saving the most lives, the forum indicated that they would choose to maintain social functioning.

ADRIANA: Accepting there will be casualties, but life has to go on.

In particular, the forum wished to uphold a life style that ensured personal independent living through continued access to essential services.

In selecting the military for the list, the forum had no particular expectation of violence, panic or disruption of society; rather, they had a favorable view of the military in a jurisdictional and humanitarian role which would assist in maintaining a structured and orderly community. The forum regarded the military as a useful resource which could assist with policing, medical and paramedical services, transport, essential services, chaplaincy and crude manpower and logistical support.

One of the forum participants (Stephanie) chose to dissent from the consensus opinion and prioritised sick people and their contacts and children in the top three. The participant questioned supporting society function if we did not also save "*the most important generation*". In the feedback interview, several of the participants commented that some provision should have been made for children. Feedback suggested that at least some of the participants were not entirely comfortable with the choices they had to make under the constraint of limited resources. For example, one participant in the feedback session said:

CATHERINE: I want the children to live and they have to carry on. They are our future.

The forum recognized that not all members of the chosen groups would need to receive the vaccine and that other measures such as quarantined workplaces might be used to protect some groups without the need for antiviral prophylaxis.

Lastly, the participants indicated their willingness to accept some increase in income tax now as a form of insurance against the threat of an emergent pandemic strain. These resources would be allocated to promoting better personal hygiene in the population, as economic aid to improve practice in the keeping and butchering of domestic fowl in developing countries, and to improve channels for informing the Australian public in a measured way and in advance about the threat. Forum members were reassured that the Government had made preparations, but were concerned that they were neither aware of these preparations nor knew how to prepare personally for a pandemic. The forum indicated it was the Government's duty to inform them of the risks and how to prepare.

### Forum 2: Consideration of quarantine and social distancing measures

#### Acceptability of quarantine and social distancing measures

Overall, the forum was divided with respect to the need for quarantine and social distancing measures. (See Table 1) Some of the participants were in favour of mandatory quarantine and social distancing measures; others considered that a policy of voluntary quarantine and social distancing should be adopted. This finding held across the full range of scenarios, with the exception of Scenario 1 for which there was unanimous agreement that quarantine was warranted, provided there was enough support for quarantined individuals.

The range of responses can be explained by three reasons that emerged in the forum. First, the forum thought that, regardless of the importance and reasonableness of quarantine and social distancing measures, not all members of the community would comply.

Second, the forum recognised that quarantine and social distancing measures would create social and emotional burdens which would influence people's willingness and capacity to comply with restrictions. For example, the forum participants expressed a range of views when discussing whether, in Scenario 2, quarantined David should be allowed to go to his wife's funeral.

TAYLA: I would sacrifice one person's happiness for the rest of the country's.

KAREN: That way if you were David -

TAYLA: I would understand.

MELISSA: He didn't see her for ten weeks...

RAELENE: My father died when I was a child ...I am very angry I wasn't allowed to go to his funeral because I was a child ... I can definitely say for David it would be a horrible experience knowing he wasn't going to be at the funeral of his wife...

JAMES: Should you be allowed to put yourself at risk knowing you could pass it on to other people? It's irresponsible...

Finally, the forum recognised that quarantine and social distancing measures, while serving some good purposes, might also undermine others, such as the importance attached to the preservation of community morale. They thought that maintaining a sense of optimism during a pandemic would be important for the wellbeing of the whole community. The forum was divided on how important such opportunities to meet would be to the maintenance of community functioning. Some participants felt more strongly than others about opportunities for sporting and religious groups to meet.

JAMES: ...If you are going to allow it [social gatherings], you have to allow all religious groups to do it. Does that include sporting clubs?

KAREN: With church, I think spiritual wellbeing is pretty important during times of crisis like pandemics.

MELISSA: The sports one, it would be an impact but not a massive huge one...It's not a necessity. It's not something you have to go to.

RAELENE: Religion is not a necessity either...

KAREN: To people who are religious it is a necessity.

Participants' views, and hence their support for social distancing measures, were influenced by their perception of the salience of the risk. They were less supportive if they considered that the risk was remote geographically. Opinion varied as to what constituted geographical remoteness; for some participants, the distance from Adelaide to Sydney was small, whereas for others Sydney was distant.

#### Supporting the community during a pandemic

The forum also considered the level of support that should be provided to those directly influenced by quarantine or social distancing measures. With the aid of the facilitator, the participants compiled a list of areas in which support could be provided; they were then asked to nominate the level of support that should be supplied. Although they had been divided on the question of whether social distancing and quarantine measures were acceptable, they were almost unanimous on the need for generous support for people affected by quarantine or social isolation. All or nearly all participants wanted at least partial income replacement (25% wanted full income replacement); deferral of debts; guaranteed return to work if leave was taken for reasons related to pandemic influenza (for example because of a quarantine restriction); food parcels; maintenance of utilities (even if bills could not be paid); and telephone counselling.

Much of the forum's discussion of financial support reflected the participants' assessment that the use of quarantine and social isolation measures in a pandemic could place job security at risk. They wanted to ensure that those members of the community who went into quarantine to protect others should not be penalised in relation to employment.

JANE: There would have to be something to allow that to happen, where there is no discrimination against the person, where they are not going to risk losing their job because they have been told "Sorry you have to go into quarantine".

How feasible it would be to compensate all income earners rather than to assign priority to those on a low income was not addressed.

#### Sanctions and enforcement

The forum was asked to nominate a range of enforcement measures and then indicate their level of support for these measures. It is important to note that the participants were assuming that adequate financial, household and psychological support would be in place before these measures would be imposed.

The participants' responses to this question were surprising. As indicated above, they had expressed a range of views about the adoption of mandatory quarantine and social distancing measures. Yet, when asked to consider how quarantine or social distancing measures should be enforced, in general they were willing to impose strict sanctions on people who did not comply. The forum was unanimous in endorsing that some level of sanction or penalty should be applied to people who violated quarantine or social distancing orders. Nearly all participants (11/12) agreed that warnings, cautions and fines for infringements were appropriate; most (9/12) considered that monitoring the activities of those who had infringed quarantine or social distancing requirements was acceptable (for example through regular telephone calls); and half thought that gaol terms for violators would be acceptable.

In summary, participants were divided on the acceptability of social distancing and quarantine measures. However, should such measures be adopted, they thought that generous financial, household and psychological support was essential. In addition, provided such support was present, the participants were in general willing to impose strict sanctions on those who violated quarantine and social distancing measures.

## Discussion

Our study has a number of implications for policy makers. First, it suggests that citizens can provide important information about community values and beliefs which may impact on the acceptability and success of pandemic containment and response strategies. Current management plans for pandemic influenza in Australia focus on health management, business continuity and information for individual households preparing for a pandemic. A number of the strategies mentioned by participants in forum 2 have already been included in Australia's pandemic influenza planning [4]. For example, there are strategies in place to support people in quarantine (through telephone support) [4] and to provide clear and consistent information [26]. There is also recognition of the psychosocial aspects of isolation, quarantine and social distancing measures [27]. In a pandemic, these strategies will be important to enhance compliance and reduce adverse psychosocial effects.

Our findings suggest, though, that these forms of support may need to be augmented. The participants considered that more comprehensive support was needed to compensate individuals for the difficulties that would arise as a result of quarantine or social distancing measures and to help assure compliance with these measures. In particular, financial support to compensate for lost income or to protect against the possibility of loss of income, and emotional support to help those in quarantine were deemed important. This type of support is not unprecedented. Job protection and income support were provided by affected countries during the SARS epidemic (summarised in [28]). Along with inability to access health care, loss of income and loss of job or business were the most frequently cited worries in a four nation survey about attitudes to quarantine [6]. Means-tested financial support for quarantine, early in a pandemic, when the case load is low and quarantine is one of the few effective tools available, may be a useful addition to pandemic management plans.

Similarly, forum 1 reflected how an informed public might react to scarcity of effective preventive resources in a pandemic. We know that, during the H1N1 pandemic influenza in Australia in 2009, stocks of antiviral drugs ran low because of demands from fearful but low risk individuals in the community. This may have been, in part, a consequence of the paucity of transparent and informed pre-pandemic debate about the use of scarce anti-viral drugs and vaccines. It would seem that this provides some support for forum 1's recommendation that the community be better informed and prepared for a pandemic.

Second, the findings give an indication of possible community responses, should quarantine and social distancing measures be mandated by law. The recent preparedness to enact emergency powers in the wake of the Influenza A (H1N1) 2009 outbreak suggests that governments at state and national levels in Australia recognise that voluntary compliance with social distancing and quarantine measures may be inadequate and that enforcement may be required. Our findings indicate that, even amongst participants who have had the opportunity to learn more about these measures, the enforcement of quarantine and social distancing measures is likely to receive a mixed response, unless supportive measures are deemed adequate. There is a need to ensure that adequate support strategies are in place to guarantee that people are not unduly disadvantaged financially by quarantine or social distancing. If adequate financial, household and psychological support is available, the findings from our forum indicate that the community may accept a range of measures, including cautions, warnings and fines.

Finally, the forums suggest that informing the public about the issues associated with pandemic planning and engaging the public in pandemic planning may mitigate opposition to the measures. The consensus of opinion for resource allocation emerging from the deliberations of forum 1 is much closer to the proposed scheme for distribution in a severe pandemic outlined in the Australian national pandemic plan (AHMPPI) [4] than that obtained from a statewide survey without prior deliberation conducted shortly before the forum [7] in which the participants prioritised the elderly and children. The priority groups selected in forum 1 fit well with the AHMPPI which, in a severe pandemic, contains provision for 'the need to maintain functioning of critical infrastructure' while prioritising pre- and post-prophylaxis for health care workers and some other occupational groups. The participants themselves identified the importance of preparing the community in advance [23]. Such preparation could lay the groundwork for effective communication in a pandemic which would moderate the potential for media overstatement and fear mongering [29,30].

It is apparent, however, that the choices made by the participants in the forums are, to a degree, uniquely Australian. For example, the inclusion of the military in the prioritised list must be considered in the context of the favourable Australian experience with the role of the Australian military in providing effective humanitarian relief in disaster situations. This finding underlines the importance of attending to local cultural beliefs and values in the development of the policy response [1].

## Conclusions

Effective implementation of pandemic plans is likely to be crucial for the successful management of a pandemic, particularly if the emergent virus is highly virulent. Countries will face particular challenges in the distribution of scarce resources in a pandemic and in implementing suitable quarantine and social isolation measures, not least because of the unique geographic, cultural, historical and social circumstances of each setting. It is difficult in this situation to develop evidence based strategies on which to base implementation. Thus, deliberative forums can provide local sources of evidence for planners and implementers of pandemic policy, and anticipate local issues which may support or impede successful implementation of plans.

However, like much public health policy, in the main pandemic planning has been carried out with little public consultation. Our study provides an exemplar for the use of deliberative process in community involvement in pandemic planning. However, like other methods, deliberative forums also suffer from drawbacks, in particular with respect to the representativeness of views presented in small forums. These issues may limit the acceptability of deliberative methods to policy-makers looking to use community views in informing policy decisions. In addition, questions have also been raised about the capacity of citizens' juries to elicit rational deliberation [31].

The findings of this study and other deliberative processes also raise questions about the usefulness of this approach in meeting the democratic ideal of including the public in decision making for public policy. We would suggest that deliberative forums such as the ones described in this study can form the basis for broader engagement with the community around the evidence through a publicity campaign to initiate broader discussion of the issues.

Understanding of community views on pandemic response strategies is relatively new. What is known is based on surveys of views about resource allocation and mitigation measures in a pandemic [6-9] and through a small number of public engagement projects [10,11]. There are similarities and differences in the findings from each of these studies, indicating that we are likely to need a range of approaches to build a comprehensive picture of community views about pandemic planning. That picture will also vary with local context, suggesting that we will also need to adjust our pandemic planning to take account of locally important factors.

The recommendations from the forums in this study suggest that the distribution of scarce resources and the implementation of quarantine and social distancing measures in Australia in a severe pandemic will be challenging, but not impossible. Implementation may be more successful if the public have more opportunities to become informed and to express their informed judgements about the issues in advance of a severe pandemic and if prudent use is made of supportive measures to assist those in quarantine or affected by social isolation measures.

## Competing interests

The authors declare that they have no competing interests.

## Authors' contributions

ABM conceived of the project, oversaw the design and execution of the project, analysed the data and drafted parts of the paper. JMS participated in the design of the project, managed the project, analysed the data and drafted the paper. WAR, RG, JRM and JEH participated in the design and execution of the project and in the revision of the paper to final draft. The FluViews team provided expertise and guidance for the FluViews project

## Pre-publication history

The pre-publication history for this paper can be accessed here:

http://www.biomedcentral.com/1471-2458/10/501/prepub
